# Valvular surgery for rheumatic heart disease in Africa: a scoping review protocol

**DOI:** 10.1097/SP9.0000000000000039

**Published:** 2025-03-20

**Authors:** ⁠Victor Oluwafemi Femi-Lawal, Victory B. Effiom, Anayo James Michael, Achanga Bill-Smith Anyinkeng, Joy Nsa Ekpenyong, Ekene Nwajei, Shubh K. Patel, Kevin R. An, Kelechi E. Okonta

**Affiliations:** a Department of Medicine and Surgery, College of Medicine, University of Ibadan, Ibadan, Oyo State, Nigeria; b Research Division, Association of Future African Cardiothoracic Surgeons, Yaounde, Cameroon; c Department of Medicine and Surgery, University of Calabar, Calabar, Nigeria; d Faculty of Health Sciences, University of Buea, Buea, Cameroon; e Temerty Faculty of Medicine, University of Toronto, Toronto, Ontario, Canada; f Division of Cardiac Surgery, Department of Surgery, University of Toronto, Ontario, Canada; g Division of Cardiothoracic Surgery, Department of Surgery, University of Port Harcourt, Nigeria

**Keywords:** Africa, rheumatic heart disease, valvular surgery

## Abstract

**Background:**

Rheumatic heart disease (RHD) remains a major health challenge in Africa, where the prevalence is notably high. Valvular surgery is a crucial procedure for managing severe RHD. However, the current state and historical trend of research on this subject in African populations is not well understood. Understanding the scope, subject matter, and quality of the literature on this topic over time is essential to inform future research and clinical practice.

**Objective:**

This paper aims to assess the current published literature to evaluate vital outcomes such as surgical outcomes, survival rates, postoperative complications, long-term quality of life, morbidities, mortalities, and barriers to valve surgery for patients with RHD in Africa.

**Methods:**

This protocol adheres to the Preferred Reporting Items for Systematic Reviews and Meta-Analyses extension for Scoping Reviews (PRISMA-ScR). An extensive search will be conducted across different databases, including PubMed, Scopus, Web of Science, Cochrane Library, African Index Medicus, and African Journals Online. Studies will be identified through keyword searches and will be reviewed against predefined inclusion and exclusion criteria by two reviewers, with a third reviewer resolving any discrepancies. A narrative synthesis will be conducted to describe the findings.

**Conclusion:**

The findings from this scoping review will provide an understanding of the current literature on valvular surgery for RHD in African contexts. This will help guide future research directions in this field.

## Introduction

Rheumatic heart disease (RHD) continues to be a significant cause of morbidity and mortality in Africa, primarily due to the ongoing prevalence of acute rheumatic fever^[1,2]^. Severe cases of RHD often necessitate surgical interventions, such as valve replacement or repair, to manage the disease effectively[3]. However, the availability of these crucial surgeries is limited in many African countries because of challenges like inadequate specialized health care, insufficient surgical training institutions, and limited resources[4]. Despite the importance of valve surgeries in managing RHD, there is a noticeable lack of comprehensive studies focusing on African populations^[5,6]^. This knowledge gap impedes efforts to improve treatment outcomes for African patients with RHD

**Highlights**

- Characterizing the clinical outcomes of valvular surgery for patients with rheumatic heart disease in Africa.
- Assessing barriers and challenges to the practice of valvular surgery for patients with RHD in Africa.
- Scoping review compliant with PRISMA-ScR guidelines.

The management of RHD through valvular surgery in Africa faces several challenges. First, the state and practice of valve repair for RHD patients in Africa are not well-documented, making it difficult to assess the quality and availability of these interventions. Additionally, access to valve surgery for RHD patients is influenced by various factors, including health care infrastructure, training of surgeons, and financial constraints, which may limit the reach of these life-saving procedures[7]. Moreover, the demographics and clinical characteristics of RHD patients requiring valve surgery, as well as the surgical techniques and interventions used, are not thoroughly understood[6]. The outcomes of these surgeries, including success rates, postoperative complications, and long-term quality of life, need to be systematically reviewed to provide a clear picture of the effectiveness of valvular surgery in this context[8].

While the highlighted challenges persist, advancements in medical imaging and image analysis are revolutionizing valve surgery for RHD. For example, image segmentation techniques play a crucial role in improving the outcomes of valve surgeries. In pre-operative planning, image segmentation helps in delineating the heart valves (e.g., mitral, aortic) and surrounding structures from imaging modalities like echocardiography, CT, or MRI. These enable detailed evaluations of valve morphology, calcifications, and leaflet motion^[9-13]^. Additionally, segmentation can generate 3D models of the heart, providing surgeons with a virtual environment to plan the procedure and select the optimal valve replacement or repair strategy^[14-16]^. Intraoperatively, image segmentation helps surgeons identify critical structures, minimizing damage to surrounding tissues and enhancing precision in valve repair or replacement. In robotic or minimally invasive surgeries, segmentation ensures proper alignment and fixation of prosthetic valves. Postoperatively, segmentation enables evaluation of the repaired or replaced valve’s performance, such as flow dynamics and residual regurgitation. Early identification of issues like paravalvular leaks, thrombosis, or endocarditis can be achieved through segmented imaging. Additionally, machine learning models trained on segmented datasets can predict outcomes, stratify patient risks, and personalize treatment pathways.

To ensure that African valvular surgery is able to advance and incorporate modern technologies and techniques, it is important to characterize the current state of the practice on the continent and provide valuable information on areas for improvement. This scoping review aims to synthesize the existing literature on valvular surgery for RHD in African settings, focusing on several key objectives. These include determining the current state and practice of valve repair, assessing access to these surgeries, and exploring the demographics and clinical characteristics of RHD patients undergoing these procedures. Additionally, the review will examine the surgical techniques and interventions used, assess the outcomes of these surgeries, and explore trends in the growth of valve surgery, advancements in techniques, and support through funding and policy initiatives. By identifying barriers to RHD valve surgery in Africa and offering recommendations for improving the delivery, access, and outcomes of these surgeries, this review seeks to contribute to a more comprehensive understanding of the field.

## Methods and analysis

This protocol adheres to the Preferred Reporting Items for Systematic Reviews and Meta-Analyses extension for Scoping Reviews (PRISMA-ScR) guidelines, to ensure that the scoping review process is entirely systematic.

### Eligibility criteria

This review will include primary research studies, such as observational studies, clinical trials, case-control studies, cohort studies, and qualitative research, as well as systematic reviews and meta-analyses focusing on valvular surgery for RHD in African populations. Only peer-reviewed sources will be considered, with exclusions for editorials, commentaries, review articles, abstracts, conference posters, and summaries.

Eligible participants include individuals diagnosed with RHD within the African continent, across all age groups. Studies exclusively focusing on non-African populations or other cardiac conditions will be excluded. Articles without accessible full texts and studies focusing exclusively on non-African populations or other cardiac conditions will also be excluded from the review.

### Search strategy

A comprehensive search strategy will be implemented across the following databases: PubMed, Scopus, Embase, Web of Science, and African Journals Online. The search terms will cover keywords related to valvular surgery, RHD, and Africa. The detailed search strategy, including specific terms and combinations, is available in the Supplement. Reference lists in the identified studies will be reviewed to capture any additional relevant literature.

### Selection of sources of evidence

Search results will be exported in RIS format and imported into the Rayyan system for screening. Two reviewers will independently screen the titles and abstracts of the studies. Any disagreements during this process will be resolved by a third reviewer. Full-text screening will be conducted on articles that meet the inclusion criteria to ensure no relevant studies are overlooked.

## Results

The primary outcome measures for this review will focus on the impact of valvular surgery on morbidity and mortality associated with RHD in African populations. Additional outcomes of interest include long-term quality of life, functional status, and patient-reported outcomes following surgical intervention.

### Data extraction

Data from the included studies will be extracted using a standardized form created by the reviewers. This form will capture key details, including study and patient demographics, the specific valves involved, the procedures performed, and clinical outcomes.

### Data charting

Findings will be aggregated in tabular form and studies will be grouped according to similarity in study type, population, type of intervention, and reported outcomes. A thematic analysis will be conducted to identify common themes, trends, and variations across the literature, allowing for a comparison of different studies and the identification of repetitive patterns. Owing to the diversity of the studies, a meta-analysis will not be performed.

## Discussion

This scoping review will present a large synthesis of the current state of the literature regarding valvular surgery for RHD in the African population. It will help in identifying existing research, highlighting which research and practice gaps must be filled, and assessing the quality of the methodology used in the literature. Further, it will inform future research endeavors toward enhancing the use and efficacy of valvular surgery for RHD in Africa.

This scoping review aims to provide a comprehensive overview of the current state of literature on valvular surgery for RHD in African populations. By synthesizing existing studies, this review will highlight the key themes, trends, and gaps in research that need to be addressed to enhance the management and outcomes of RHD through surgical interventions. Additionally, it will evaluate the quality of methodologies used in the literature, providing a basis for determining whether there is sufficient evidence to justify the initiation of a systematic review.

Understanding the current landscape of valvular surgery for RHD in Africa is crucial, given the high burden of the disease and the limited access to specialized surgical care in many regions. Studies such as Watkins *et al*[1]. have documented the significant burden of RHD in Africa, emphasizing the need for improved surgical interventions to manage the disease effectively. Furthermore, the limited availability of trained cardiac surgeons and the necessary infrastructure, as highlighted by Zilla *et al*[17], poses a significant challenge to the effective management of RHD in these settings. The disparity in access to cardiac surgery across different regions of Africa has also been noted by Sliwa *et al*[18], who underscore the need for targeted efforts to improve health care delivery in low-resource settings. This review will consider these challenges and identify potential strategies to overcome them, thereby informing future research and policy efforts aimed at enhancing the availability and effectiveness of valvular surgery in African health care systems.

Ultimately, this scoping review seeks to contribute to the ongoing global health dialogue on improving cardiac care in low-resource settings. By identifying areas where further research is needed and proposing recommendations for enhancing surgical interventions, this review will support efforts to improve patient outcomes and reduce the burden of RHD in Africa. It also aims to encourage the development of targeted interventions and partnerships that can address the specific needs of African populations affected by this disease.

### Strengths and limitations

This review strengthens a thorough search strategy and compliance with the PRISMA-ScR guidelines, ensuring methodological rigor and transparency. However, potential limitations include language bias since only English-language studies will be considered, and the possibility of missing relevant studies not indexed in the selected databases. Additionally, gaps in existing research may limit the ability to draw comprehensive conclusions.

## Data Availability

No datasets were generated or analyzed in the current study.
